# Clinical Characteristics and Treatment Outcomes of Alcohol Withdrawal Syndrome in Adolescents and Young Adults

**DOI:** 10.1016/j.jaacop.2024.01.012

**Published:** 2024-03-25

**Authors:** Hayrunnisa Unlu, Asmaa Yehia, Sherif El-Gayar, Amogh Havanur, Farha Deceus, Samantha J. Brown, Sarah B. Umar, Paul E. Croarkin, Terry D. Schneekloth, Osama A. Abulseoud

**Affiliations:** a Mayo Clinic Arizona, Phoenix, Arizona; b Mansoura University, Mansoura, Egypt; c Graduate School of Biomedical Sciences, Mayo Clinic College of Medicine, Phoenix, Arizona; d Alix School of Medicine at Mayo Clinic, Phoenix, Arizona; e Mayo Clinic Rochester, Rochester, Minnesota

**Keywords:** adolescents, alcohol withdrawal syndrome, delirium tremens, suicidality, substance misuse

## Abstract

**Objective:**

Despite the high prevalence of underage drinking, little is known about alcohol withdrawal syndrome (AWS) in adolescents and young adults. The aim of this study is to characterize AWS in this population.

**Method:**

We conducted a retrospective chart review of all hospital admissions with the Clinical Institute Withdrawal Assessment of Alcohol Scale, Revised (CIWA-Ar) protocol at Mayo Clinic between June 2019 and June 2022.

**Results:**

We identified a total of 10,220 patients with 16,338 hospital admissions where CIWA-Ar protocol was implemented. Within this cohort, 130 patients (70 male and 60 female; 1.3% of all patients) with 148 admissions (0.9% of all admissions) were under 21 years of age. A total of 44% of patients (n = 65) presented with suicidal ideations, and 26% (n = 40) had suicide attempts. In all, 22 (n = 33) required admission to the intensive care unit. The median length of stay in the intensive care unit was 32.8 hours. The median peak CIWA-Ar score was 9 (minimum-maximum = 4-39, interquartile range = 7), and the median time from hospital admission to peak CIWA-Ar score was 9.4 hours (minimum-maximum = 0.1-126, interquartile range = 20.2). A total of 40% of patients (n = 59) received benzodiazepines, whereas 20% (n = 31) required antipsychotics. Three patients (2%) developed delirium tremens, and 5 episodes of alcohol withdrawal seizures (3.4%) were observed. No deaths were reported during hospitalization. However, over the subsequent follow-up period from 2019 to 2023, the all-cause post-hospitalization mortality rate was 3% (n = 4) within 1.6 (±0.6) years.

**Conclusion:**

These data suggest that adolescents and young adults presenting for treatment of AWS are at risk for morbidity and mortality due to suicidality and withdrawal complications such as withdrawal seizures and delirium tremens. Further studies should evaluate the underlying social and neurobiological predictors of vulnerability and resilience in this age group.

## Plain language summary

A study of adolescents and young adults experiencing alcohol withdrawal (AW) at Mayo Clinic identified 130 patients under 21 years old among 10,220 patients with AW (1.3% of the total) and 148 admissions among 16,338 admissions (0.9% of all AW admissions). Results revealed concerning trends in these minors: 44% presented with suicidal thoughts, 26% attempted suicide, and 22% required treatment in the intensive care unit. Although no in-hospital deaths were recorded, 4 patients (3%) experienced all-cause mortality within 1.6 years after discharge, but the causes of death remained unknown. These findings highlight the health risks of underage drinking and emphasize the need for further research.

Underage drinking is a serious public health problem,^1^, ^2^, ^3^, ^4^, ^5^, ^6^ with estimates that youth who consume alcohol below the legal drinking age spend at least $22.5 billion (of $116.2 billion in total expenditure) on alcohol purchases annually^7^^,^^8^ and consume approximately 11.7% of all alcoholic drinks sold in the United States market in 2011.^9^ Indeed, several large-scale epidemiological studies document the high prevalence of alcohol drinking in adolescents and young adults. Recent data from the Monitoring the Future Survey in 2022 (n = 31,438) showed that 15% of 8^th^ graders, 31% of 10^th^ graders, and 52% of 12^th^ graders reported alcohol use in the past year, whereas the lifetime prevalence was 23.1% for 8^th^ graders, 41.1% for 10^th^ graders, and 61.6% for 12^th^ graders.^10^ Moreover, the 2021 National Survey on Drug Use and Health revealed that 15.1% (n = 5.9 million people) consumed alcohol in the past month, and 8.3% (n = 3.2 million people) engaged in binge drinking (drinking ≥5 drinks for male individuals or ≥4 drinks for female individuals on the same occasion on at least 1 day in the past 30 days) and 1.6% (n = 613,000) heavy drinking (drinking ≥5 drinks for male individuals and ≥4 drinks for female individuals on the same occasion on 5 or more days in the past 30 days) among individuals 12 to 20 years of age. The same survey reported that 894,000 youth 12 to 17 years of age (3.4% of this population) had an alcohol use disorder (AUD) diagnosis in the past year.^11^ In addition, results from school surveys indicate that alcohol use commonly starts before the age of 15 years, and the prevalence of alcohol use among 15-year-old students ranges between 50% and 70%.^12^ This high rate of underage drinking is associated with poor school performance and involvement in health risk behaviors,^13^ drunk driving,^14^ alcohol intoxication–related fatal car crashes,^15^ and other complications.^13^^,^^16^, ^17^, ^18^, ^19^, ^20^, ^21^

Alcohol withdrawal syndrome (AWS) is a potentially life-threatening complication of heavy drinking^22^^,^^23^ and is associated with withdrawal seizures,^24^, ^25^, ^26^ delirium tremens,^27^^,^^28^ and mortality.^29^, ^30^, ^31^ To date, clinical literature on AWS has been limited to adult populations,^22^^,^^31^, ^32^, ^33^, ^34^, ^35^ with only a few case reports in adolescents,^36^, ^37^, ^38^ suggesting that AWS is rare in this population. Arguably, the short duration of alcohol consumption in adolescents and young adults could be associated with less risk for AWS.^39^ In addition, findings from animal models of alcohol withdrawal are inclined toward the rare sighting of AWS in adolescents. For example, during acute ethanol withdrawal, adolescent male Sprague-Dawley rats did not exhibit manifestations of anxiety in the elevated plus maze^40^^,^^41^ or in the social behavior interaction task,^42^ which could suggest an increased propensity for binge drinking among adolescents. However, adolescent male C57BL/6J mice bingers did not show increased anxiety in the marble-burying anxiety test.^43^ On the other hand, findings by Montagud-Romero *et al.* opposed the notion of AWS-resistance in adolescents. They exposed pregnant C57BL/6 female mice to binge drinking from gestation to weaning and assessed alcohol-seeking behavior in the adolescent male offspring, using the 2-bottle choice and oral self-administration paradigms.^44^ The adolescents’ offspring showed higher alcohol intake and, interestingly, enhanced anxiety-like responses during acute alcohol withdrawal, specifically in prenatal and lactational alcohol-exposed mice.^44^ Nevertheless, methodological issues limit the interpretation of these results including scoring individual manifestations of withdrawal such as anxiety^40^^,^^41^^,^^43^ by using short-term observations (15 seconds) at various time points after cessation of ethanol administration^45^^,^^46^ or by collapsing all withdrawal signs into 1 score.^47^ Neither of these methods could capture the complexity of human AWS, in which different manifestations emerge at different time windows over an extended period after the last drink.^48^ As such, the current preclinical data on adolescent and young adult AWS may not reflect the full clinical syndrome, and the question remains whether the high prevalence of binge drinking in adolescents and young adults is associated with AWS in this population.

The aim of this study was to identify the prevalence, clinical features, and treatment outcomes of youth who consume alcohol below the legal drinking age with AWS among all hospital admissions placed on the Clinical Institute Withdrawal Assessment of Alcohol Scale, Revised (CIWA-Ar) protocol^49^ across several hospitals in a large regional health system. We hypothesized that at least 1% of all admissions placed in CIWA-Ar would be adolescents and young adults with varying degrees of severity and complications.

## Method

### Patients and Procedures

The study was approved by the Institutional Review Board of Mayo Clinic (ID#22-008591) in compliance with all international and institutional research standards. We collected the electronic medical records of all admissions for patients who were placed under CIWA-Ar protocol for alcohol withdrawal at Mayo Clinic Health System during the interval from June 2019 through June 2022. The Mayo Clinic Health System is a family of clinics, hospitals, and other healthcare facilities with a physical presence in 44 communities in 4 regions of southern Minnesota, western Wisconsin, and northern Iowa. The majority (>90%) of patients are White and non-Hispanic. Pediatric clinics treat patients up to 18 years of age. However, adolescents in this study presented to the emergency department and were subsequently admitted to the child and adolescent psychiatric unit. Young persons 18 to 20 years of age were admitted to the internal medicine or adult psychiatric service. Youth who consumed alcohol below the legal drinking age at the time of the first CIWA-Ar protocol–related admission were included in this study. All patient data were initially obtained from an electronic data extraction by senior data analysts. Following this, 3 independent authors (AH, FGD, and SB) verified the electronic medical record and performed data cleaning. Next, the lead author (HU) manually cross-checked individual data points by reviewing patients’ charts, including providers’ notes, progress notes, admission and discharge notes, and laboratory results. Admission diagnoses were collected from the providers’ notes of the first encounter. Active problem lists and active problems noted by providers were used to report medical and psychiatric comorbidities and substance use profiles. Any history of suicide attempts mentioned in the patient chart or listed in the problem list, at any time point prior to the admission date, were included as history of suicide attempt. The CIWA-Ar scale is a 10-item survey used to quantify the severity of withdrawal manifestations: (1) nausea and vomiting; (2) tremors; (3) sweating; (4) anxiety; (5) agitation; (6) tactile disturbances; (7) auditory disturbances; (8) visual disturbances; (9) headache; and (10) disorientation or clouding of the sensorium. Scores on the CIWA-Ar range from 0 to 67 points.^49^ Patients are placed on CIWA-Ar protocol when they report AWS manifestations or cessation of heavy drinking. The symptom-triggered lorazepam administration is a treatment approach within the CIWA-Ar protocol that involves administering lorazepam based on the severity of alcohol withdrawal symptoms as measured by CIWA-Ar scores. Patients who score from 0 to 9 do not receive lorazepam treatment; patients who score 10 to 12 receive 1 mg of lorazepam orally or intravenously (PO/IV); those who a score 13 or 14 receive 2 mg of lorazepam PO/IV; those who score 15 to 17 receive 3 mg of lorazepam PO/IV; and those who score of 18 or greater receive 4 mg of lorazepam PO/IV. AWS symptoms are reassessed 30 minutes after lorazepam administration for further dosing. This approach has been associated with lowering benzodiazepine doses, which yields less sedation, shorter hospitalizations, fewer adverse reactions, and a decreased risk of respiratory depression in comparison to fixed-dose benzodiazepine treatment.^50^

The first identified encounter for each patient was included in the analysis for demographics and comorbidities in cases of multiple encounters for a single patient. For all episodes (n = 148), patient data included demographics, reason for hospital admission, comorbid medical and psychiatric conditions, each CIWA-Ar assessment from admission until discharge, hospital course including intensive care unit (ICU) admissions, benzodiazepine and other treatments (anticonvulsants, antipsychotics, antibiotics, thiamine, dexmedetomidine), and withdrawal complications. Administered benzodiazepine and antipsychotic doses were converted to lorazepam and haloperidol equivalent doses, respectively.^51^^,^^52^

### Statistical Analysis

The normality of continuous variables was assessed using the Shapiro–Wilk test. Data are presented as mean ± SD if normally distributed; as median, interquartile range (IQR), and range if not normally distributed; and categorical data are expressed as percentages. To compare continuous variables between male and female patients, we used the Student *t* test for normally distributed data and the Mann–Whitney *U* test for non-parametric data. Categorical variables were compared using the χ^2^ test or Fisher exact test. Regression analysis was used to examine the association between peak the CIWA-Ar score and the total lorazepam dose administered. We excluded 6 patients from the hospital and ICU LOS analysis because these 6 patients required long-term hospitalization for reasons other than AWS treatment (multiple traumas, valproic acid toxicity, and eclampsia). A *p* value of ≤.05 was considered statistically significant. All analyses were performed using standard SPSS software (IBM Corp.).

## Results

### Demographics

Between June 2019 and June 2022, we identified a total of 10,220 patients with 16,338 hospital admissions based on the implementation of the CIWA-Ar protocol for AWS. Within this cohort, 130 patients (1.3% of all patients) with 148 admissions (0.9% of all admissions) were younger than 21 years of age. In all, 11% of male (n = 8) of male and 5% of female (n = 3) patients had more than 1 hospital admission during the study interval. Male and female patients were approximately equally represented (53.8%, n = 70 male patients; 46.2%, n = 60, female patients). The median age of the group was 19 years (minimum-maximum = 14-20 years, interquartile range [IQR] = 2). Most patients were White (76.9%, n = 100) and non-Hispanic (85.4%. n = 111). Approximately two-thirds (65.4%, n = 85) of all patients did not report their level of education, whereas slightly more than one-fourth (28.5%, n = 37) were in high school and 6.1% (n = 8) were in college. In addition, 5.4% of the patients (n = 7) were homeless. The demographic characteristics did not differ significantly between male and female patients (Table 1).

**Table 1:** Patient Demographics

Characteristic	All Patients (n = 130)	Male patients (n = 70, 53.8%)	Female patients (n = 60, 46.2%)	*p*
Age, y, median (IQR)	19 (2)	19 (2)	19 (2)	.3
Race				
African American	16 (12.3%)	6 (8.6%)	10 (16.7%)	.65
Other	8 (6.2%)	6 (8.6%)	2 (3.3%)	.86
Unknown	6 (4.6%)	4 (5.7%)	2 (3.3%)	.99
White	100 (76.9%)	54 (77.1%)	46 (76.7%)	.99
Ethnicity				
Non-Hispanic	111 (85.4%)	60 (85.7%)	51 (85.0%)	.99
Hispanic	14 (10.8%)	7 (10%)	7 (11.7%)	.99
Unknown	5 (3.8%)	3 (4.3%)	2 (3.3%)	.99
Employment status				
Student	24 (18.5%)	9 (12.9%)	15 (25.0%)	.3
Employed	44 (33.8%)	27 (38.6%)	17 (28.3%)	.87
Unemployed	35 (26.9%)	19 (27.1%)	16 (26.7%)	.99
Unknown	27 (20.8%)	15 (21.4%)	12 (20%)	.99
Educational status				
Less than 12th grade or GED	16 (12.3%)	8 (11.4%)	8 (13.3%)	.99
12th grade	21 (16.2%)	14 (20.0%)	7 (11.7%)	.99
Some college	8 (6.1%)	1 (1.4%)	7 (11.7%)	.08
Unknown	85 (65.4%)	47 (67.1%)	38 (63.3%)	.99
Homelessness	7 (5.4%)	5 (7.1%)	2 (3.3%)	.4
BMI, kg/m^2^, median (IQR)	23.3 (7.7)	22.3 (7.4)	24.1 (7.9)	.4
BMI category, no. (%)				
BMI <18.5 kg/m^2^	10 (7.7%)	5 (7.1%)	5 (8.3%)	.8
BMI 18.5-24.9 kg/m^2^	61 (46.9%)	35 (50%)	26 (43.3%)	.4
BMI 25-29.9 kg/m^2^	33 (25.4%)	17 (24.3%)	16 (26.7%)	.7
BMI 30-39.9 kg/m^2^	14 (10.8%)	8 (11.4%)	6 (10%)	.8
BMI ≥40 kg/m^2^	2 (1.5%)	0 (0%)	2 (3.3%)	.1
Unknown	10 (7.7%)	5 (7.1%)	5 (8.3%)	.8

Note: BMI = body mass index; GED = General Equivalency Diploma.

### Reasons for Hospitalization

All patients sought initial medical care in the emergency room (ER) and subsequently required admission to various departments for further treatment, most commonly to a psychiatric unit (90%, n = 117). The primary reason for hospitalization in adolescents and young adults experiencing AWS was suicidality, encompassing suicidal ideations and attempts, which was reported in 70.8% of all admissions (n = 105). Specifically, 44.6% of patients (n = 65) presented to the ER with suicidal ideations, and 26.2% (n = 40) had made a suicide attempt. We examined suicidality rates in the presence and absence of alcohol intoxication, defined as blood alcohol concentration (BAC) ≥0.08% (n = 67), and found no difference between the 2 conditions (suicidality rate with BAC ≥ vs BAC < 0.08%: 70.1%, n = 47, vs 71.6%, n = 58). All drug overdose incidents were suicide attempts and accounted for 24.3% (n = 36) of all admission diagnoses and 88.2% (n = 30) of all suicide attempts. The second most common reason for admission was alcohol intoxication (45.4%, n = 67), followed by alcohol withdrawal (16.9%, n = 22), altered mental status (excluding the post-ictal state, 16.9%, n = 22), and trauma (8.46%, n = 11). Furthermore, 4.6% (n = 6) of patients who were initially admitted for non–alcohol-related conditions, such as pyelonephritis (n = 2), diabetic ketoacidosis (n = 2), and skin infections (n = 2), subsequently developed AWS and required the implementation of the CIWA-Ar protocol during the hospital stay.

With regard to reasons for hospital admissions, there were notable differences between male and female patients. A higher proportion of female patients presented with suicide attempts (38.5% of female patients, n = 25, vs 18.1% of male patients (n = 15) , *p* = .01), whereas male patients were significantly more likely to be admitted with altered mental status (27.1% of male patients, n = 19. vs 5% of female patients, n = 3; *p* = .0008) and visual or auditory disturbances due to AWS (12% of male patients, n = 10, vs 1.5% of female patients, n = 1, *p* = .02) (Table 2).

**Table 2:** Reasons for Hospital Admission in Study Patients

Reason	All hospitalizations (n = 148)	Male patients, (n = 83, 56%)	Female patients (n = 65, (44%)	*p*
Suicidality				
Any	105 (70.9%)	51 (61.4%)	54 (83.1%)	.006∗
Ideation	65 (43.9%)	36 (43.4%)	29 (44.6%)	.99
Attempt				
Total	40 (27%)	15 (18.1%)	25 (38.5%)	.009∗
Drug overdose	36 (24.3%)	13 (15.7%)	23 (35.4%)	.007∗
Other^a^	4 (2.7%)	2 (2.4%)	2 (3.1%)	.99
Alcohol intoxication, BAC ≥0.08%	67 (45.3%)	38 (45.8%)	29 (44.6%)	.99
Altered mental status^b^	27 (18.2%)	24 (28.9%)	3 (4.6%)	<.001∗
Trauma	11 (7.4%)	8 (9.6%)	3 (4.6%)	.34
Seizure	11 (7.4%)	6 (7.2%)	5 (7.7%)	.99
Admission withdrawal symptoms				
Visual or auditory disturbance	11 (7.4%)	10 (12%)	1 (1.5%)	.02∗
Tremor	6 (4%)	3 (3.6%)	3 (4.6%)	.99
Anxiety	6 (4%)	3 (3.6%)	3 (4.6%)	.99
Vomiting	4 (2.7%)	2 (2.4%)	2 (3.1%)	.99
Agitation	3 (2%)	1 (1.2%)	2 (3.1%)	.58
Pyelonephritis	2 (1.3%)	0 (0%)	2 (3.1%)	.19
Diabetic ketoacidosis	2 (1.3%)	2 (2.4%)	0 (0%)	.5
Skin infection	2 (1.3%)	0 (0%)	2 (3.1%)	.19
Abdominal pain				
All	2 (1.3%)	2 (2.4%)	0 (0%)	.5
Cholecystitis	1 (0.7%)	1 (1.2%)	0 (0%)	.99
Perforated peptic ulcer	1 (0.7%)	1 (1.2%)	0 (0%)	.99
Pneumomediastinum	1 (0.7%)	1 (1.2%)	0 (0%)	.99

Note: BAC = blood alcohol concentration.

∗*p* < .05.

a Jumping into traffic (n = 1), jumping from height (n = 1), cutting wrists (n = 1), poisoning by CO_2_ (n = 1).

b Other than postictal state

### Comorbid Medical Conditions

Over half of the patients (58.5%, n = 76) had comorbid medical conditions at the time of hospital admission. The most prevalent comorbidity was gastrointestinal (GI) disorders, including gastritis, gastroesophageal reflux disease (GERD), and peptic ulcers, affecting 15.4% of patients (n = 20). Trauma accounted for 18.5% (n = 11), fractures for 6.9% (n = 9), and ear, nose, and throat (ENT) disorders for 6.2% of the cases (n = 8). In terms of sex differences, female patients were more frequently diagnosed with asthma (10% of female, n = 6, vs 0% of male patients, *p* = .009) and sexually transmitted infections (6.7% of female, n = 4, vs 0% of male patients, *p* = .04) (Table 3).

**Table 3:** Comorbid Medical and Psychiatric Conditions of Study Patients

	Condition	All patients (n = 130)	Male patients (n = 70, 53.8%)	Female patients (n = 60, 46.2%)	*p*
Medical	Any	76 (58.5%)	43 (51.8%)	33 (50.8%)	.99
	Gastrointestinal disorder	20 (15.4%)	10 (12%)	10 (15.4%)	.63
	Trauma	11 (8.5%)	7 (8.4%)	4 (6.2%)	.75
	Fracture	9 (6.9%)	6 (7.2%)	3 (4.6%)	.73
	Ear, nose, and throat disorder	8 (6.2%)	7 (8.4%)	1 (1.5%)	.08
	Cardiovascular disorder	7 (5.4%)	6 (7.2%)	1 (1.5%)	.13
	Pain condition	7 (5.4%)	3 (3.6%)	4 (6.2%)	.7
	Chronic headache including migraine	7 (5.4%)	3 (3.6%)	4 (6.2%)	.7
	Asthma	6 (4.6%)	0 (0%)	6 (9.2%)	.006∗
	Seizure disorder	6 (4.6%)	5 (6%)	1 (1.5%)	.23
	Anemia or malnutrition	6 (4.6%)	1 (1.2%)	5 (7.7%)	.09
	Dermatological condition including psoriasis	5 (3.8%)	5 (6%)	0 (0%)	.06
	Leukocytosis, other infections including aspiration pneumonia	5 (3.8%)	3 (3.6%)	2 (3.1%)	.99
	Renal disorder	5 (3.8%)	2 (2.4%)	3 (4.6%)	.65
	Syncopal episode	5 (3.8%)	3 (3.6%)	2 (3.1%)	.99
	SARS-CoV-2–positive test result	5 (3.8%)	2 (2.4%)	3 (4.6%)	.65
	Diabetes	4 (3.1%)	3 (3.6%)	1 (1.5%)	.63
	Sexually transmitted disease	4 (3.1%)	0 (0%)	4 (6.2%)	.03∗
	Respiratory failure	2 (1.5%)	2 (2.4%)	0 (0%)	.5
	Gynecological disorder	2 (1.5%)	NA	2 (3.1%)	.2
	Fetal alcohol syndrome	1 (0.8%)	1 (1.2%)	0 (0.0%)	.99
	Thyroid disorder	1 (0.8%)	0 (0%)	1 (1.5%)	.44
	Sleep apnea	1 (0.8%)	1 (1.2%)	0 (0%)	.99
	Rhabdomyolysis	1 (0.8%)	1 (1.2%)	0 (0%)	.99
Psychiatric	Any^a^	117 (90%)	61 (73.5%)	56 (86.2%)	.07
	Depression	72 (55.4%)	35 (42.2%)	37 (56.9%)	.09
	Previous suicide attempt	48 (36.9%)	19 (22.9%)	29 (44.6%)	.008∗
	Previous suicidal ideation	44 (33.8%)	24 (28.9%)	20 (30.8%)	.8
	Generalized anxiety disorder	39 (30%)	17 (20.5%)	22 (33.8%)	.09
	History of altered mental status	31 (23.8%)	21 (25.3%)	10 (15.4%)	.16
	Attention-deficit/hyperactivity disorder	29 (22.3%)	21 (25.3%)	8 (12.3%)	.06
	Post-traumatic stress disorder	15 (11.5%)	4 (4.8%)	11 (16.9%)	.02∗
	Bipolar disorder	11 (8.5%)	5 (6%)	6 (9.2%)	.53
	Insomnia or hypersomnia	6 (4.6%)	1 (1.2%)	5 (7.7%)	.08
	Eating disorder	3 (2.3%)	0 (0%)	3 (4.6%)	.08
	Gender dysphoria	4 (3%)	1 (1.2%)	3 (4.6%)	.32
	Childhood abuse	3 (2.3%)	0 (0%)	3 (4.6%)	.08
	Conversion disorder	3 (2.3%)	1 (1.2%)	2 (3.1%)	.58
	Disruptive behavior disorder	2 (1.5%)	2 (2.4%)	0 (0%)	.5
	Autism spectrum disorder	2 (1.5%)	2 (2.4%)	0 (0%)	.5
	Schizoaffective disorder	2 (1.5%)	2 (2.4%)	0 (0%)	.5
Substance use	Any	90 (69.2%)	48 (68.6%)	42 (70%)	.99
	Cannabis	74 (56.9%)	39 (55.7%)	35 (58.3%)	.85
	Tobacco	35 (26.9%)	12 (17.1%)	23 (38.3%)	.01∗
	Cocaine or methamphetamine	26 (20%)	16 (22.9%)	10 (16.7%)	.51
	Benzodiazepine	18 (13.8%)	8 (11.4%)	10 (16.7%)	.38
	Opioid	7 (5.4%)	5 (7.1%)	2 (3.3%)	.45
	Hallucinogen	3 (2.3%)	3 (4.3%)	0 (0%)	.25
	Barbiturate	1 (0.8%)	1 (1.4%)	0 (0%)	.99

**Note:**

∗*p* < .05.

a Other than substance use.

### Comorbid Psychiatric Conditions and Substance Use Profiles

Almost all patients (90%, n = 117) had non–substance use comorbid psychiatric disorders. With the inclusion of co-morbid substance use, 94.6% (n = 123) had a comorbid psychiatric and/or substance use disorder. Depression emerged as the most prevalent comorbid psychiatric disorder, affecting 55.4% of all patients (n = 72). Significantly more female than male patients had a history of previous suicide attempts (44.6% of female patients, n = 29, vs 22.9% of male patients, n = 19, *p* = .008) and post-traumatic stress disorder (PTSD) (16.9% of female patients, n = 11, vs 4.8% of male patients, n = 4, *p* = .02). Comorbid substance use (in addition to alcohol) was documented in 69.2% of the patients (n = 90). Among the misused substances, cannabis had the highest prevalence at 56.9% (n = 74), followed by tobacco (26.9%, n = 35), cocaine and/or methamphetamine (20%, n = 26), and benzodiazepines (13.8%, n = 18). No sex difference was observed in substance use profiles, except for the higher prevalence of tobacco use among female patients (38.3% of female patients, n = 23, vs 17.1% of male patients, n = 12, *p* = .01) (Table 3).

### Hospital Course Including ICU Admissions and AWS Complications

The median blood alcohol concentration at the time of hospital admission was 80 mg/dL (minimum-maximum = 0-404, IQR = 174), with no difference between male and female patients. In addition, there was no difference in BAC between suicidal (n = 105) and non-suicidal (n = 43) patients (median [IQR] BAC level: 81 [174.8] mg/dL in suicidal group vs 67 [181.5] mg/dL in non-suicidal group, *p* = .7). The median hospital LOS duration was 68.8 hours (minimum-maximum = 2.2-330.2, IQR = 81.9). About one-fifth of the patients (22.3%, n = 33) were admitted to the intensive care unit (ICU). The median ICU LOS was 29.3 hours (minimum-maximum = 7.4-77, IQR = 22.5), with no difference between male and female patients. There was no significant difference in the hospital LOS between patients admitted to the ICU and those in non-ICU settings (median = 70.9 [75.5] hours for ICU patients, 68.7 [87.9] hours for non-ICU patients, *p* = .8). Similarly, patients receiving benzodiazepine treatment required a shorter hospital LOS, but the difference was not statistically significant (median = 64.5 [84.7] hours for benzodiazepine-treated patients, 70 [82.4] hours for non–benzodiazepine treated patients, *p* = .7). However, patients requiring benzodiazepine treatment had a longer ICU LOS (median [IQR] = 37 [15.4] hours for benzodiazepine-treated patients, 18.9 [21.1] hours for non–benzodiazepine-treated patients, *p* = .008). Three patients (2%) developed DT; all 3 of these patients were male. Of the 148 episodes of alcohol withdrawal, 5 alcohol withdrawal seizures (3.4%) were observed in 3 patients (2 male and 1 female) (Table 4).

**Table 4:** Hospital Course of Study Patients

Hospital course	All hospitalizations (n = 148)	Male patients (n = 83, 56%)	Female patients (n = 65, 44%)	*p*
Hospital LOS, h, median (IQR), min-max	68.8 (112.8), 2.2-330.2	61.8 (74.7)	77.3 (89)	.24
ICU admissions, n (%)	33 (22.3%)	20 (24.1%)	13 (20%)	.69
ICU LOS, median (IQR), min-max	29.3 (22.5), 7.4-77	30 (16.7)	26.8 (15.7)	.58
Blood alcohol concentration (n = 117), mg/dL, median (IQR), min-max	80 (174), 0-404	80 (189), 0-404	80.5 (145.3), 0-338	.66
Patients with total CIWA-Ar score ≥4, n (%)	96 (64.9%)	52 (62.7%)	44 (67.7%)	.6
Peak total CIWA-Ar score, median (IQR), min-max)	9 (7), 4-39	9 (10.7)	8.5 (6)	.87
Time from admission to peak total CIWA-Ar score, h	9.4 (*R* = 0.08-126; IQR = 20.2)	7.8 (19.5), *R* = 0.0 8-123.9	10.1 (23.1), *R* = 0.08-126	.08
Benzodiazepine treatment over the entire hospital LOS				
Patients who received treatment, n (%)	59 (39.9%)	36 (43.4%)	23 (35.4%)	.4
Median (IQR), min-max (lorazepam equivalent in mg)	3 (7), 0.5-48.8	4 (6.7)	2 (7)	.24
Benzodiazepine treatment over the first 24 h of admission				
n (%)	58 (39.2%)	35 (42.2%)	23 (35.4%)	.49
Median (IQR), min-max (lorazepam equivalent in mg)	2 (4.2), 0.5-43	2 (5)	2 (4)	.88
Thiamine treatment^a^				
n (%)	123 (83.1%)	69 (83.1%)	54 (83.1%)	.99
Median (IQR) total dose (mg)	300 (300)	300 (300)	300 (400)	.4
Antipsychotic treatment^a^				
n (%)	31 (20.9%)	16 (19.3%)	15 (23.1%)	.68
Median (IQR) dose (haloperidol equivalent) (mg)	7.5 (9.5)	5 (8.6)	10 (10.7)	.57
Anticonvulsant treatment^a^^,^^b^				
n (%)	13 (8.8%)	9 (10.9%)	4 (6.2%)	.39
Antibiotic treatment^a^^,^^c^				
n (%)	13 (8.8%)	7 (8.4%)	6 (9.2%)	.99
Dexmedetomidine treatment^a^				
n (%)	11 (7.4%)	5 (6%)	6 (9.2%)	.53
Median (IQR) (μg/kg/h)	3.3 (20.9)	3.3 (16.6)	2.65 (37.3)	.79
AWS complication				
Delirium tremens	3 (2%)	3 (3.6%)	0 (0%)	.2
Alcohol withdrawal seizures	5 (3.4%)	4 (4.8%)	1 (1.5%)	.4
Elevated liver enzymes^d^	16 (17%)	11 (22%)	5 (11.4%)	.3
Post-hospitalization mortality				
n (%)	4 (3.1%)	3 (4.3%)	1 (1.7%)	.6
Mean (±SD) duration between first hospitalization and death, y	1.6 (0.6)	2.2 (0)	1.4 (0.5)	.3
Laboratory results				
Alkaline phosphatase (n = 89, M/F = 48/41), median (IQR), min-max	80 (36.5), 10-274	71 (33), 10-138	86 (39.5), 50-274	.01∗
ALT (n = 94, M/F = 50/44), median (IQR), min-max	19 (14.5), 7-219	17.5 (10.7), 8-219	21.5 (25.7), 7-217	.005∗
AST (n = 94, M/F = 51/43), median (IQR), min-max	27 (15.5), 11-214	22 (12), 11-212	30 (17), 15-214	.002∗
Platelet count (n = 118, M/F = 64/54), mean (SD)	272 (70.3)	284.6 (64.3)	261.3 (45)	.02∗

Note: AWS = alcohol withdrawal syndrome; ALT = alanine transaminase; AST = aspartate transaminase; F = female patients; ICU = intensive care unit; IQR = interquartile range; LOS = length of stay; M = male patients; min-max = minimum-maximum.

∗*p* < .05.

a Over the entire hospital LOS.

b Anticonvulsants include lamotrigine (n = 7), levetiracetam (n = 3), phenobarbital (n = 2), zonisamide (n = 1).

c Antibiotics include ceftriaxone (n = 7), piperacillin-tazobactam (n = 3), and vancomycin (n = 3).

d ALT >55 U/L and/or AST >48 U/L (percentage was calculated in 94 patients who had laboratory values of ALT and AST).

### AWS Symptoms and CIWA-Ar Scores

The most frequently reported AWS symptom was anxiety (65.5%, n = 97), followed by nausea and vomiting (25%, n = 37), headache (23.6%, n = 35), paroxysmal sweats (23.6%, n = 35), and tremors (22.3%, n = 33). There were no sex differences in AWS symptoms.

CIWA-Ar was implemented within the first hour of hospitalization in 24.3% of cases (n = 36). Within the first 6 hours of hospitalization, 51.5% of hospitalizations (n = 81) were under the CIWA-Ar protocol. CIWA-Ar was implemented within 6 to 24 hours of hospitalization in 33.8% of cases (n = 50). Finally, CIWA-Ar was implemented after the first day of hospitalization in 11.5% of cases (n = 17). Of 148 hospitalizations, 64.9% of the patients (n = 96) scored ≥4 on the CIWA-Ar scale. The median peak CIWA-Ar score was 9 (minimum-maximum = 4-39, IQR = 7), with no difference between male and female patients. As expected, patients who received benzodiazepine treatment had higher CIWA-Ar scores (median [IQR] for benzodiazepine-treated vs non–benzodiazepine-treated patients: 12 [10] vs 6 [5], *p* = 0.0001). In addition, there was no difference in the peak CIWA-Ar score between suicidal (n = 105) and non-suicidal (n = 43) patients (median peak CIWA-Ar score: 8.5 [7] in suicidal group vs 9 [7.3] in non-suicidal group, *p* = .6).

**Figure 1:**
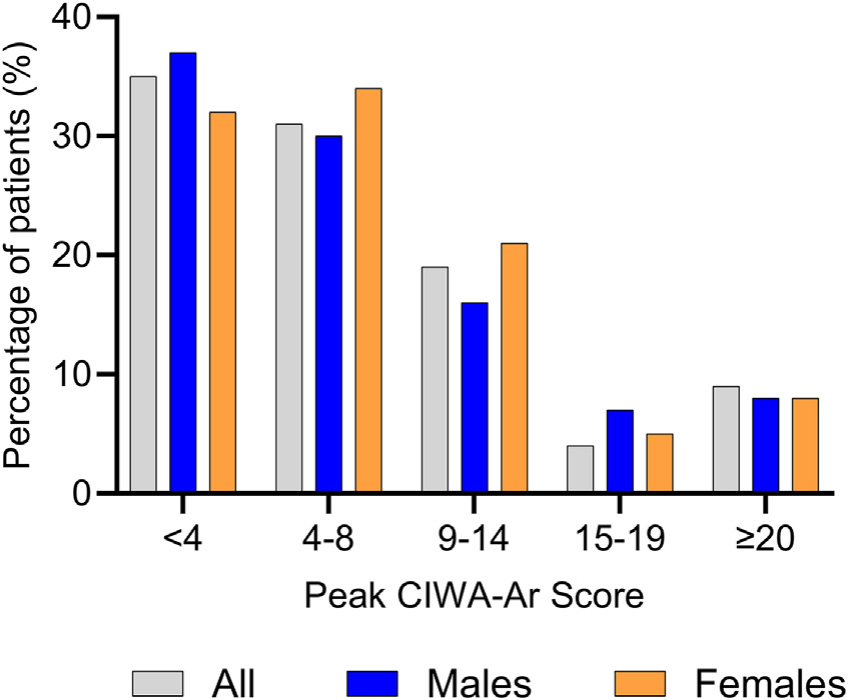
Histogram Showing the Percentage of Patients at Different Peak Score Groups ***Note:****CIWA-Ar = Clinical Institute Withdrawal Assessment of Alcohol Scale,Revised.*

The median time between hospital admission and maximum AWS symptomatology, determined by peak CIWA-Ar score, was 9.4 hours (minimum-maximum = 0.08-126, IQR = 20.2). Half of all patients (50%, n = 74) reached peak CIWA-Ar score during the first 24 hours of hospitalization, regardless of sex (Figure 1). Patients with severe conditions that required ICU admission took longer to reach peak CIWA-Ar score (median [IQR] for ICU-admitted vs non–ICU-admitted patients: 21.2 [38] vs 8 [17.8] hours, *p* = .01) (Table 4).

### Laboratory Results

The median serum alkaline phosphatase (ALP) concentration was 80 IU/L (minimum-maximum = 10-274, IQR = 36.5), whereas the median serum alanine aminotransferase (ALT) concentration was 19 (minimum-maximum = 7-219, IQR = 14.5) and aspartate transaminase (AST) was 27 (mimimum-maximum = 11-214, IQR = 15.5). Liver enzymes were significantly higher in male than in female patients (median [IQR] of male vs female patients (ALP: 86 [39.6] vs 71 [33], *p* = .01; ALT: 21.5 [25.8] vs 17.5 [10.8], *p* = 0.05; AST: 30 [17] vs 22 [12], *p* = .02). The mean (SD) platelet count was significantly lower in male than in female patients (male vs female patients: 261.3 [44.9] vs 284.6 [64.3], *p* = .02) (Table 4 and Table S1 and Table S2, available online).

### AWS Treatment

**Figure 2:**
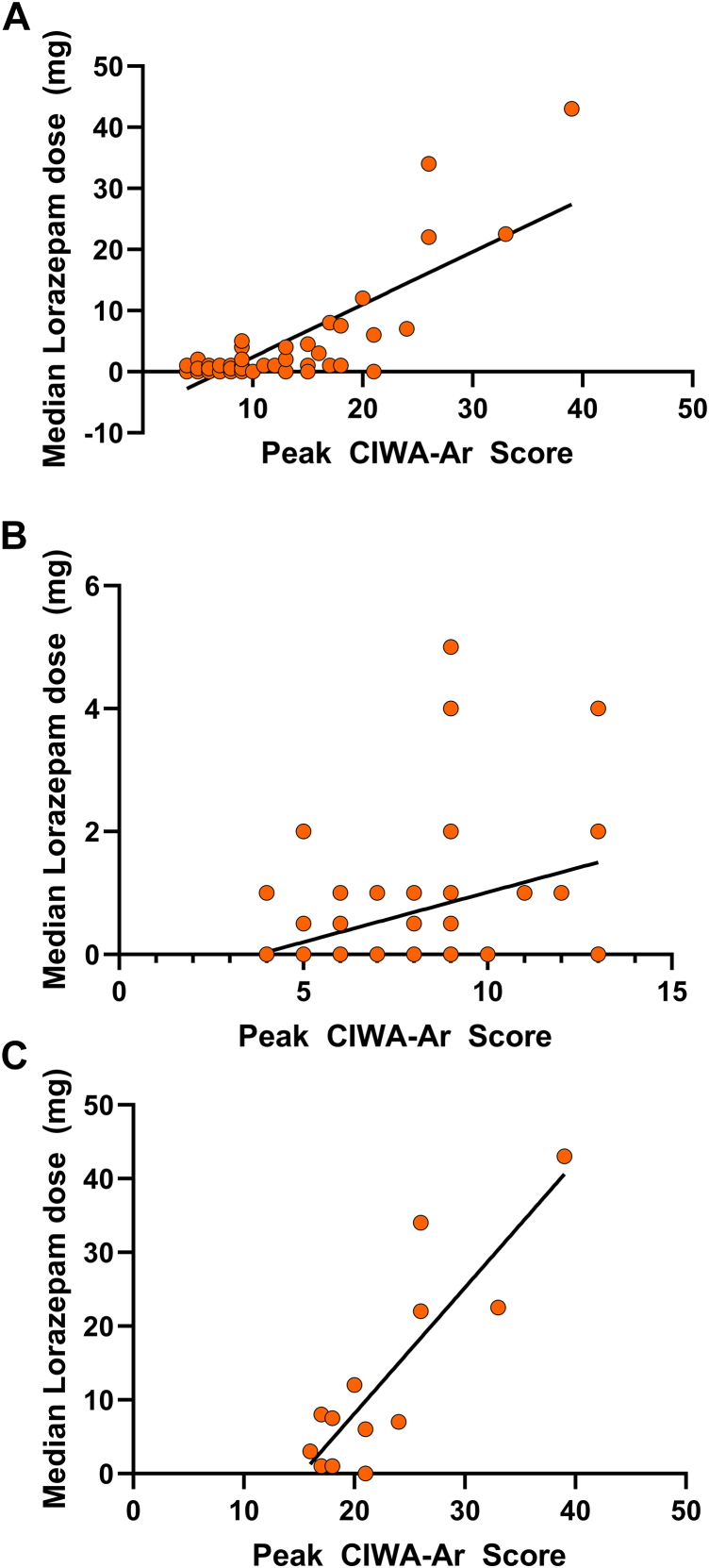
Association Between Peak Clinical Institute Withdrawal Assessment of Alcohol Scale, Revised (CIWA-Ar) Score and Total Lorazepam Dose Administered in the First 24 Hours of Hospitalization **Note:** (A) All patients; (B) patients with CIWA-Ar score ≥15; and (C) patients with CIWA-Ar score <15.

Among the patients placed on CIWA-Ar monitoring, 83.1% (n = 123) received oral thiamine; the median thiamine dose over the hospital LOS was 300 mg (IQR = 300). As many as 39.9% of the patients (n = 59) required benzodiazepine treatment. The median dose of benzodiazepine over the whole course of the hospital stay (lorazepam equivalent) was 3 mg (minimum-maximum = 0.5-48.8, IQR = 7). Almost all of these patients (98.3%, n = 58) received benzodiazepine during the first day of hospitalization. The median dose of benzodiazepine on the first day of hospitalization was 2 mg (minimum-maximum = 0.5-43, IQR = 4.3). A significant positive correlation between peak CIWA-Ar score and total lorazepam dose (in milligrams [mg]) administered during the first 24 hours of admission was observed (all peak CIWA-Ar scores *F*_1,71_ = 140.6, *p* < .0001) (Figure 2A) in both severe (peak CIWA-Ar scores >15 *F*_1,11_ = 31.83, *p* = .0002) (Figure 2B) and mild-to-moderate withdrawal (peak CIWA-Ar scores ≤15 *F*_1,55_ = 10.18, *p* = .002) (Figure 2C), but not on the second day of admission (*F*_1,16_ = 0.1027, *p* = .7).

In addition, 20.9% of the patients (n = 31) required antipsychotic treatment for agitation or worsening psychosis. The median dose of antipsychotic treatment (haloperidol equivalent) over the whole course of the hospital stay was 7.5 mg (minimum-maximum = 1-26 mg, IQR = 9.5). All 3 DT patients received antipsychotics at a higher median (IQR) dose of 10 (12.5) mg compared to non–DT agitated patients (5 [9] mg, *p* = .3) (Table 4).

### Mortality

No deaths were observed during hospitalization. Over the subsequent follow-up period from 2019 to 2023, the mortality rate was 3% (n = 4; 3 male patients and 1 female patient). Among these patients, only 1 patient had a medical comorbidity (diabetes mellitus type 1). Regarding admission diagnosis, 2 were admitted because of suicidality, 1 because of trauma, and 1 because of diabetic ketoacidosis. The mean (±SD) duration between the first hospitalization and death was 1.6 (0.6) years. The reasons for death were not reported.

### Comparison of Adolescents (14-17 Years) and Young Adults (18-20 Years)

We divided our patients into 2 subgroups for further comparison based on age: 13.1% of the patients (n = 17) belonged to the 14- to 17-year adolescent age group, whereas 86.9% (n = 113) were in the 18- to 20-year young adult age group. Adolescents had longer hospital stays compared to young adults (116.8 [70.3] hours vs 64.1 [76.9] hours, *p* < .02). The percentage of young adults receiving thiamine was higher than adolescents (86.8%, n = 112, vs 57.9%, n = 11, *p* < .005). No significant differences were observed between the groups regarding ICU stay, AWS complications, time to peak CIWA-Ar score, peak CIWA-Ar score, laboratory values, and other treatment outcomes (Table S3, available online).

### Effect of the COVID-19 Pandemic

We compared patients who were admitted before the COVID-19 pandemic (admissions between June 2019 and February 2020) and during the pandemic (March 2020 to June 2022). Within cohort, 26.2% of the patients (n = 34) and 24.3% of the hospital admissions were before the pandemic. There was no difference between groups regarding hospital course, ICU stay, AWS complications, time to peak CIWA-Ar score and the peak CIWA-Ar score, laboratory values, and treatment outcomes (Table S4, available online).

## Discussion

To the best of our knowledge, this is the first study to evaluate the prevalence, clinical characteristics, and treatment outcome of alcohol withdrawal syndrome (AWS) in adolescents and young adults. Youth under the age of 21 years who consumed alcohol and were placed on the CIWA-Ar protocol for AWS represented 1.3% of all patients admitted to the hospital and placed on the CIWA-Ar protocol. Our study cohort had an equal representation of male and female patients, who also had very high rates of psychiatric (90%) and medical (58%) comorbidities. Almost three-fourths of all cases had suicide attempts or ideations at the time of hospitalization. Even though most of our patients did not score high on CIWA-Ar, we observed high rates of ICU admission (22.3%), benzodiazepine treatment (40%), antipsychotic administration (21%), alcohol withdrawal seizure (3.4%), alcohol withdrawal DT (2%), and post-hospitalization mortality (3%). These results highlight critical public health concerns associated with underage drinking, and challenge the concept of adolescent and young adult resistance to alcohol withdrawal.

There was a strikingly high rate of suicide attempts (27%) and ideations (44%) in these young patients. A survey conducted by the Substance Abuse and Mental Health Services Administration (SAMHSA) in 2021 reported that 12.7% (3.3 million) of surveyed adolescents 12 to 17 years of age reported suicidal ideation in the year prior to the survey, whereas 5.9% (1.5 million) made suicide plans and 3.4% (892,000) attempted suicide.^11^ The 8-fold higher (27% vs 3.4%) rate of those in our study, as compared to the community rate, likely reflects the presentation to the ER with both suicidal ideation or attempts in combination with recent alcohol use, justifying their hospital admission for monitoring and treatment. Nonetheless, the rate of suicide attempts in our patients is comparable to that of adults with alcohol intoxication or severe alcohol use disorder.^53^, ^54^, ^55^, ^56^, ^57^ Completed suicide is a leading cause of adolescent mortality.^11^^,^^58^^,^^59^ We had 4 patients (3%) who died within a median of 1.6 years following hospital admission and treatment for AWS, speaking to the high-risk status of this group. Although the reasons for death were not mentioned in the medical records, suicide may have played a role in this high mortality rate. As such, further research focused on predictors of suicidality in adolescents and young adults with alcohol use is urgently needed.

In our study, we investigated the occurrence of alcohol withdrawal DT and seizures among patients. Of the 148 admissions, 3 patients (2%) developed DT, and we observed 5 alcohol withdrawal seizures in 3 patients (3.4%). It is worth noting that, apart from the reported 3 DT cases in this study, there is only 1 documented case report of DT in a 9-year-old child.^60^ Surprisingly, there is a lack of data regarding alcohol withdrawal seizures among adolescents and young adults. To date, our study has reported the highest rates of DT and alcohol withdrawal seizures among adolescents and young adults. These rates are comparable to those in adult studies. Our recent review^61^ reported that the collective DT rate was 25% (1,792/7,147) for male adults and 17% (307/1,924) for female adults. The collective alcohol withdrawal seizure rate was 7.4% (294/3,974) for male adults and 8.5% (82/966) for female adults. Regardless of the rate of DT and withdrawal seizures, our documented findings of DT and withdrawal seizures in youth less than 21 years of age who consume alcohol are critical information for clinicians to foster their monitoring and treatment of AWS in at-risk adolescent and young adult patients.

Upon admission, the blood alcohol concentration (BAC) was measured in 90% of cases (n = 117), and nearly half of all cases exhibited intoxication, with a median BAC of 80 mg/dL (minimum-maximum: 0-404). Our BAC results are higher than those in a recent study from Holland (19 mg/dL) in 482 adolescents <18 years of age who were admitted for acute alcohol intoxication.^62^ On the other hand, a study from Sweden in adolescents 15 to 20 years of age (n = 3,513) who were arrested for driving under the influence of alcohol reported a much higher BAC (132 mg/dL).^63^ These variations in BAC could reflect different ages (<18 vs up to 20 years of age), different study settings, and the timing of BAC measurement after the last drink. Moreover, BAC results did not differ between male and female patients in our study or in the Swedish study. These findings support emergency department screening for alcohol use in both male and female adolescents and young adults, particularly those presenting with suicidal ideation or attempt, and monitoring for withdrawal manifestations when heavy drinking is reported or suspected.

In our study, the median hospital length of stay (LOS) was 2.8 days (IQR = 4.7). When comparing our results to previous studies conducted in adults, it is important to note that the mean (SD) hospital LOS in those studies ranged from 8 (5) to 11.5 (9.1) days.^26^^,^^33^^,^^64^ Although it is difficult to compare mean and median, we acknowledge that the hospital LOS can be influenced by medical comorbidities^64^ or AWS severity.^26^^,^^64^ About 58% of our patients had comorbid medical conditions, and 10% required ICU admission. The ICU admission rate in our study is also comparable to that of adults, which reportedly ranges from 3.5% to 16%,^26^^,^^32^ suggesting a similar degree of AWS severity between adults and adolescents or young adults.

Half of our patients reached the peak CIWA-Ar score during the first 24 hours (median [IQR] of 9.4 [20.2] hours) of hospitalization. In adults, minor alcohol withdrawal typically begins around 6 hours following the last drink and reaches its peak between 18 and 24 hours, whereas moderate to severe alcohol withdrawal tends to peak later and could last for up to 6 days.^65^ The shorter time to peak in our cohort could be attributed to a shorter duration of alcohol drinking or a faster rate of alcohol metabolism in adolescents and young adults compared to older adults.^66^ These findings support the utility of the CIWA-Ar in adolescent alcohol withdrawal, the probable benefits from the earlier aggressive medication of withdrawal symptoms, and the relatively rapid resolution of symptoms facilitating briefer LOS than in patients ≥21 years of age.

The median total peak CIWA-Ar score in our study was 9 (mimimum-maximum = 4-39, IQR = 7). One study in adults reported that the mean (SD) CIWA-Ar score was 4.5 (3.4).^67^ Another study reported a median Clinical Institute Withdrawal Assessment for Alcohol (CIWA-A)^68^ (not CIWA-Ar) score ranging from 23 to 30.^33^ These results are not comparable because the CIWA-A scale is a 15-item scale with scores ranging from 0 to 86, whereas the CIWA-Ar scale is a 10-item scale with a score ranging from 0 to 67.^49^ As such, the question remains whether AWS severity or time to peak withdrawal depends on age despite some,^26^^,^^39^^,^^69^ but not all,^22^^,^^33^^,^^64^^,^^70^^,^^71^ studies suggesting increased severity with increased age. In support of this concept, the median lorazepam dose in our study during the first day of admission was 2 mg (IQR = 4.3), which is lower than the mean lorazepam dose equivalent range of 8.5 mg to 11 mg (diazepam: 42.5 [36.6] mg to 55.7 [27.3] mg]) in a study in adults with AWS.^33^ A longer period of intensive drinking and the kindling phenomenon, in which repeated episodes of alcohol withdrawal may lead to increased severity or sensitivity of withdrawal symptoms, are essential considerations in understanding AWS across developmental stages as individuals progress into young adulthood, potentially affecting the severity and recurrence of withdrawal symptoms.^33^^,^^72^ One study reported that although older patients (>60 years of age) started intensive drinking 11 years later than younger patients (<30 years of age), they engaged in harmful alcohol consumption for a longer duration (an average of 18 years). However, the study noted that younger patients had a higher incidence of past withdrawal seizures compared to older patients. On the other hand, older patients had prolonged hospital stays, and the correlation between the severity of AWS symptoms and the number of previous alcohol withdrawal episodes was highest among the older group.^33^ To understand age differences and developmental frameworks, large-scale and prospective studies with head-to-head comparisons of AWS manifestations between different ages are needed.

The cohort did not show a sex gap among adolescents and young adults with AWS. This finding is consistent with recent data in adult populations showing a robust increase in the prevalence of excessive alcohol drinking among women^73^, ^74^, ^75^, ^76^, ^77^; women exhibit more sensitivity to alcohol, progressing to alcohol-related diseases at lower drinking levels compared to men.^78^, ^79^, ^80^ However, we observed some sex differences in initial presentation of AWS and its complications. Female patients presented with suicide attempts, specifically with drug overdose, whereas male patients presented mostly with altered mental status and visual or auditory disturbances. In our study, 3 of 3 DT cases in our study were male, 2 of 3 experiencing withdrawal seizures were male, and 3 of the 4 patients who died were male. All of these case findings need replication in large national data sets before drawing conclusions about the potential sex difference in AWS among adolescents and young adults.

Recent data demonstrates that COVID-19 pandemic had an impact on adolescent alcohol consumption,^81^^,^^82^ but there are no reports on its effect on AWS in this age group. On the other hand, the literature reports increased AWS among adult hospitalized patients during the pandemic.^83^ It is possible that the change in alcohol drinking patterns during the COVID-19 pandemic confounded our results. However, we did not find any difference between the pre- and during-pandemic hospitalizations. Future studies are needed to understand the effect of the COVID-19 pandemic on adolescent alcohol withdrawal.

There are several strengths and notable limitations of this retrospective study. First, our cohort was defined by a reliable objective measure (the CIWA-Ar protocol) with documented records of the AWS phenotype, clinical course, and treatment outcome. In addition, male and female individuals were equally represented, which made it possible to examine potential sex differences. The implementation of the CIWA-Ar protocol relies on the clinical judgment of health care professionals, and it can vary in different hospital settings. It is possible that some patients may have experienced a delayed initiation of the CIWA-Ar protocol until their symptoms had advanced. As such, the results of this study may not reflect the full spectrum of the AWS. However, we observed that 64.9% of the patients scored below 4 on the CIWA-Ar, indicating that even individuals with mild withdrawal symptoms were included in the CIWA-Ar protocol. Among other limitations, we could not conduct prospective data collection. The study population of 130 individuals may not be representative of general adolescent and young adult hospitalized populations. In fact, these patients had high rates of comorbid depression (55.4%), attention-deficit/hyperactivity disorder (ADHD; 22.3%), post-traumatic stress disorder (PTSD; 11.5%), and bipolar disorder (8.5%). These rates are several-fold higher than the rates of depression (11.3%),^84^ ADHD (6.8%),^85^ PTSD (3%),^86^ or bipolar disorder (1%)^87^ in the general adolescent population. Notably, there was a pregnant adolescent who presented with both eclampsia and alcohol withdrawal. Similarly, 70% of our patients presented with substance use in addition to alcohol consumption, with cannabis being the most commonly used substance (56.9%), followed by tobacco (26.9%), stimulants (cocaine or methamphetamine; 20%), and benzodiazepine (13.8%). Again, these rates are higher than the published rates for adolescents in general (cannabis use: 33%,^88^ tobacco use: 13.7%,^89^ methamphetamine use: 4.1%,^20^ and benzodiazepine or sedative hypnotic use: 5.8%^90^). All medical and psychiatric diagnoses and substance use profiles were collected from the active problem list and providers’ notes. Providers issued these diagnoses based on the clinical presentation, as the patients were not enrolled in a prospective study in which the Structured Clinical Diagnostic Interview (SCID) for *DSM* or other screening tool was used. Mortality data following discharge were acquired from the electronic medical record, but it is important to acknowledge the potential for missing data for patients who discontinued their follow-up at Mayo Clinic. Studies have shown that apart from Asian American subgroups, racial and ethnic minorities in the United States are at higher risk for experiencing symptoms of alcohol dependence, negative outcomes from drinking, and alcohol-related health issues and deaths compared to the White population.^91^, ^92^, ^93^ Therefore, our study’s demographic composition may not fully represent the adolescent and young adult population facing AWS. In addition, the literature reported that there are disparities in the quality and accessibility of alcohol treatment services based on race, ethnicity, and socioeconomic status.^92^, ^93^, ^94^ Our study’s results highlight these disparities, as our sample comprised predominantly White and non-Hispanic patients who received treatment at the Mayo Clinic, reflecting a demographic with high-quality care but limited diversity. By raising awareness of social injustice and health inequality, we advocate for more comprehensive and equitable mental health services to mitigate the existing inequalities and to promote better outcomes. Future studies involving diverse populations are needed to better understand and to effectively address these disparities, as well as to gain a comprehensive understanding of the clinical presentation of AWS in adolescents and young adults. Although our sample is the largest to date, it included a small sample size and encompassed only a 3-year window; future studies would ideally include large populations of hospitalized youth below the legal drinking age who consume alcohol, with an extended follow-up duration. In addition, we acknowledge the limitations posed by a wide age range (14-20 years) within our study; future studies targeting specific age brackets would offer a deeper understanding. Moreover, the sampling method, starting with all hospitalizations with documented CIWA-Ar protocol instead of starting with all patients with documented AWS diagnosis in the problem list used in this study, could potentially introduce bias into the sample, affecting the generalizability of our findings. We have observed t3 cases of DT and 5 cases of alcohol withdrawal seizures among 148 hospitalizations for AWS. Other than these few cases, almost all other CIWA-Ar scores reflected mild to moderate withdrawal. As such, we do not have enough numbers of severe AWS to identify risk factors of severity of withdrawal. In our study, a small group of patients 14 to 17 years of age (n = 17) highlighted the need for larger studies to specifically examine AWS in this age group. The time and amount of the last alcohol consumption and the average number of drinks per drinking day, or the number of drinking days per week, all remain unknown because of the retrospective nature of the study. Future studies are needed to better understand these aspects of AWS in adolescents and young adults.

In conclusion, the results of this study highlight the possibility and public health concern of AWS, including severe manifestations such as DT and seizures among adolescents and young adults. This cohort also demonstrated the potential lethal combination of underage drinking and suicidal ideation or suicide attempts among adolescent and young adult hospital admissions. The detailed description of the medical and psychiatric comorbidities and withdrawal manifestations in this cohort of 148 admissions carries significant clinical relevance and raises awareness for the importance of future studies.

## CRediT authorship contribution statement

**Hayrunnisa Unlu:** Writing – review & editing, Writing – original draft, Project administration, Methodology, Investigation, Formal analysis. **Asmaa Yehia:** Writing – review & editing, Data curation. **Sherif El-Gayar:** Data curation, Writing – review & editing. **Amogh Havanur:** Data curation, Writing – review & editing. **Farha Deceus:** Data curation, Writing – review & editing. **Samantha J. Brown:** Data curation, Writing – review & editing. **Sarah B. Umar:** Data curation, Writing – review & editing. **Paul E. Croarkin:** Writing – review & editing, Data curation. **Terry D. Schneekloth:** Writing – review & editing, Data curation. **Osama A. Abulseoud:** Writing – review & editing, Writing – original draft, Supervision, Methodology, Investigation, Formal analysis, Conceptualization.
