# Supplementary material for: Clinical Characteristics and Treatment Outcomes of Alcohol Withdrawal Syndrome in Adolescents and Young Adults
Source: JAACAP Open. 2024 Mar 25;3(2):216–31. doi: 10.1016/j.jaacop.2024.01.012 (PMC12166939; doi:10.1016/j.jaacop.2024.01.012)
Supplement: Supplemental Material [file mmc1.docx]

**Table S1: CBC with differential for all, for males vs females**

|  | Hemoglobin  (n=118, M/F=64/54)  [Mean (SD)] | Hematocrit  (n=118, M/F=64/54)  [Mean (SD)] | Red blood cell (n=118, M/F=64/54)  [Mean (SD)] | MCV  (n=118, M/F=64/54)  [Mean (SD)] | RDW  (n=118, M/F=64/54)  [Mean (SD)] | Platelet  (n=118, M/F=64/54)  [Mean (SD)] | Leukocyte (n=116, M/F=63/53)  [Mean (SD)] | Neutrophil (n=102, M/F=57/45)  [Mean (SD)] | Lymphocyte (n=102, M/F=57/45)  [Mean (SD)] | Monocyte (n=102, M/F=57/45)  [Mean (SD)] | Eosinophil  (n=102, M/F=57/45)  [Median (IQR)] | Basophil  (n=102, M/F= 57/45)  [Median (IQR)] |
| --- | --- | --- | --- | --- | --- | --- | --- | --- | --- | --- | --- | --- |
| All | 14.4 (1.6) | 42.7 (4.5) | 4.8 (0.5) | 89.4 (5.6) | 12.7 (1.4) | 272 (70.3) | 8.2 (3.1) | 5.1 (2.4) | 1.9 (1) | 0.6 (0.4) | 0.08 (0.1) | 0.04 (0.03) |
| Males | 15.3 (1.2) | 45.1 (3.6) | 5 (0.4) | 89.1 (4.38) | 12.7 (0.81) | 261.25 (45) | 9 (2.8) | 5.7 (2.4) | 2.2 (1) | 0.8 (0.3) | 0.03 (0.05) | 0.03 (0.01) |
| Females | 13.3 (1.3) | 39.9 (3.7) | 4.5 (0.5) | 87.9 (5.6) | 13.4 (1.9) | 284.64 (64.32) | 8.4 (2.2) | 5.3 (2.1) | 2.2 (0.9) | 0.6 (0.3) | 0.1 ( | 0.03 ( |
| p-value | <0.001* | <0.001* | <0.001* | 0.19 | 0.01* | 0.02* | 0.15 | 0.39 | 0.92 | 0.003* | 0.7 | 0.39 |

CBC=Complete blood count

F=Females

M=Males

∗p < 0.05

**Table S2: Comprehensive Metabolic Panel for all, for males vs females**

|  | K  (n=121, M/F=64/ 57)  [Mean (SD)] | Na  (n=121, M/F=64/ 57)  [Mean (SD)] | Cl  (n=121, M/F=64/ 57)  [Mean (SD)] | Mg (n=20, M/F=11/ 9)  [Mean (SD)] | HCO3 (n=112, M/F=60/ 52)  [Mean (SD)] | Anion gap (n=120, M/F=64/ 56)  [Mean (SD)] | Ca  (n=117, M/F=64/ 53)  [Mean (SD)] | Glucose  (n=116, M/F=61/ 55)  [Mean (SD)] | Protein  (n=84, M/F=45/39)  [Mean (SD)] | Albumin  (n=85, M/F=46/ 39)  [Mean (SD)] | Bilirubin (n=85, M/F=46/ 39)  [Mean (SD)] | BUN  (n=121, M/F= 64/57)  [Median (IQR)] | Cr (n=121, M/F=64/ 57)  [Mean (SD)] | ALP  (n=89, M/F= 48/41)  [Median (IQR)] | ALT  (n=94, M/F= 50/44)  [Median (IQR)] | AST (n=94, M/F= 51/43)  [Median (IQR)] | TSH (n=68, M/F=35/33)  [Mean (SD)] |
| --- | --- | --- | --- | --- | --- | --- | --- | --- | --- | --- | --- | --- | --- | --- | --- | --- | --- |
| All | 3.8 (0.5) | 140.1 (3.6) | 103 (4.5) | 2 (0.3) | 23 (4.7) | 14 (4.7) | 9.2 (0.5) | 101 (21.7) | 7.4 (1) | 4.6 (0.6) | 0.4 (0.35) | 10 (5) | 0.8 (0.2) | 80 (36.5), 10-274 | 19 (14.5),7-219 | 27 (15.5), 11-214 | 1.7(1.45) |
| Males | 3.81 (0.4) | 140.1 (3.45) | 102.7 (3.81) | 2.1 (0.17) | 22.94 (3.71) | 14.57 (4.1) | 9.3 (0.55) | 105.5 (23.2) | 7.4 (0/75) | 4.7 (0.59) | 0.65 (0.69) | 12 (3) | 0.9 (0.18) | 86 (39.55), 50-274 | 21.5 (25.75), 7-217 | 30 (17), 15-214 | 1.69 (0.8) |
| Females | 3.8 (0.35) | 140 (3.81) | 104.1 (4.1) | 2.24 (1.05) | 22.96 (3.21) | 12.91 (2.91) | 9.1 (0.48) | 99.9 (16.4) | 7.3 (0.75) | 4.4 (0.52) | 0.4 (0.2) | 10 () | 0.74 (0.14) | 71 (33), 10-138 | 17.5 (10.75), 8-219 | 22 (12), 11-212 | 1.92 (1.37) |
| P-value | 0.89 | 0.91 | 0.053 | 0.65 | 0.98 | 0.01* | 0.06 | 0.13 | 0.56 | 0.005* | 0.03* | 0.01* | <0.001* | 0.01* | 0.005* | 0.002* | 0.19 |

ALT= Alanine transaminase

AST= Aspartate transaminase

ALP= Alkaline phosphatase

BUN= Blood urea nitrogen

Cr=Creatinine

F=Females

M=Males

TSH= Thyroid Stimulating Hormone

∗p < 0.05

**Table S3: Hospital Course in different age groups (14-17 years vs 18-20 years)**

|  | | 14-17 years | 18-20 years | p-value |
| --- | --- | --- | --- | --- |
| Sex (n (%), M/F) | | 9 (52.9%) /8 (47.1%) | 61 (54%) /52 (46%) | 0.99 |
| Number of Patients | | 17 (13.1%) | 113 (86.9%) | 0.8 |
| Number of Hospital Admissions | | 19 (12.8%) | 129 (87.2%) | 0.8 |
| Hospital LOS (hours) [Median (IQR)] | | 116.8 (70.3) | 64.1 (76.9) | 0.02* |
| No. (%) of ICU admissions | | 1 (5.3%) | 32 (24.8%) | 0.08 |
| ICU LOS (hours) [Median (IQR)] | | 21 | 29.3 (22.6) | N/A |
| Blood alcohol concentration (mg/dL) (n=117, 14-17/18-20=13/104) [Median (IQR), Min-Max] | | 47 (117.5) | 81 (180.8) | 0.4 |
| Peak CIWA-Ar Scores [Median (IQR)] | | 9 (10) | 8 (7) | 0.6 |
| Patients with total CIWA-Ar score ≥4 [No. (%)] | | 12 (63.2%) | 84 (65.1%) | 0.99 |
| Time (hours) from admission to peak total CIWA-Ar Score | | 9.4 (14.3) | 5.2 (20.7) | 0.2 |
| Benzodiazepine (Lorazepam equivalent) treatment over the whole hospital LOS | No. (%) | 7 (36.8%) | 52 (40.3%) | 0.99 |
|  | Median (IQR) (lorazepam equivalent in mg) | 2 (2) | 4 (9.9) | 0.2 |
| Thiamine treatment ^a^ | No. (%) | 11 (57.9%) | 112 (86.8%) | 0.005* |
|  | Median (IQR) dose (mg) | 300 (300) | 300 (300) | 0.1 |
| Antipsychotic (Haloperidol equivalent) treatment | No. (%) | 1 (5.3%) | 30 (23.3%) | 0.2 |
|  | Median (IQR) dose (mg) | 2.5 | 8.3 (8.4) | N/A |
| Anticonvulsant treatment ^a, b^ | No. (%) | 0 (0%) | 13 (10.1%) | 0.2 |
| Antibiotic treatment ^a, c^ | No. (%) | 0 (0%) | 13 (10.1%) | 0.2 |
| Dexmedetomidine treatment ^a^ | No. (%) | 1 (5.3%) | 10 (7.8%) | 0.5 |
|  | Median (IQR) dose (mcg/kg/hr) | 23 | 3.3 (15.3) | N/A |
| AWS complications | Delirium Tremens | 0 (0%) | 3 (2.3%) | 0.99 |
|  | Alcohol withdrawal seizures | 0 (0%) | 5 (3.9%) | 0.99 |
|  | Elevated liver enzymes ^d^ | 0 (0%) | 16 (12.4%) | 0.2 |
| Post-hospitalization mortality | No. (%) | 0 (0%) | 4 (3.1%) | 0.99 |
|  | Mean (±SD) duration (years) between 1^st^ hospitalization and death | N/A | 1.6 (0.6) | N/A |
| Laboratory results | Alkaline phosphatase (n=89, 14-17/18-20= 8/81), [Median (IQR)] | 85.5 (85.1) | 79 (35) | 0.4 |
|  | ALT (n=94, 14-17/18-20=8/86), [Median (IQR)] | 18 (16.2) | 19 (15.8) | 0.4 |
|  | AST (n=94, 14-17/18-20=8/86), [Median (IQR)] | 23.5 (6.2) | 27 (17.3) | 0.3 |
|  | Platelet count (n=118, 14-17/18-20=9/109), [Median (IQR)] | 287 (84) | 270 (69) | 0.1 |

AWS= Alcohol withdrawal syndrome

ALT= Alanine transaminase

AST= Aspartate transaminase

ICU=Intensive care unit

LOS=Length of stay

N/A=Not applicable

^a^ Over the whole hospital LOS

^b^ Anticonvulsants include Lamotrigine, Levetiracetam, Phenobarbital, Zonisamide

^c^ Antibiotics include Ceftriaxone, Levofloxacin, Piperacillin-Tazobactam, Vancomycin

^d^ ALT> 55 U/L and/or AST> 48 U/L (the percentage was calculated in a total of 94 patients who had laboratory values of ALT and AST).

∗p < 0.05

**Table S4: Hospital Course in patients who were admitted to the hospital before the COVID-19 pandemic (June 2019-Feb 2020) and during the pandemic (March 2020-March 2022)**

|  | | Pre-Pandemic | During Pandemic | p-value |
| --- | --- | --- | --- | --- |
| Age | | 19.6 (2.1) | 19.6 (1.5) | 0.3 |
| Sex (n (%), M/F) | | 19 (55.9%) /15 (44.1%) | 51 (53.1%) /45 (46.9%) | 0.8 |
| Number of Patients | | 34 (26.2%) | 96 (73.8%) | 0.8 |
| Number of Hospital Admissions | | 36 (24.3%) | 112 (76.7%) | 0.8 |
| Hospital LOS (hours) [Median (IQR)] | | 64.4 (94.5) | 70.1 (81.5) | 0.6 |
| No. (%) of ICU admissions | | 10 (27.8%) | 23 (20.5%) | 0.4 |
| ICU LOS (hours) [Mean (SD)] | | 25.3 (21.5) | 29.3 (25.6) | 0.3 |
| Blood alcohol concentration (mg/dL) (n=117, Pre pandemic/Post pandemic=33/84) [Median (IQR), Min-Max] | | 67 (184) | 83 (175) | 0.9 |
| Peak CIWA-Ar Scores [Median (IQR)] | | 7 (5) | 9 (9) | 0.1 |
| Patients with total CIWA-Ar score ≥4 [No. (%)] | | 19 (52.8%) | 77 (68.8%) | 0.1 |
| Time (hours) from admission to peak total CIWA-Ar Score | | 7.1 (14.2) | 9.9 (21.9) | 0.2 |
| Benzodiazepine (Lorazepam equivalent) treatment over the whole hospital LOS | No. (%) | 11 (30.6%) | 48 (42.9%) | 0.2 |
|  | Median (IQR) (lorazepam equivalent in mg) | 6.5 (5.5) | 9.5 (8.5) | 0.2 |
| Thiamine treatment ^a^ | No. (%) | 28 (77.8%) | 95 (84.8%) | 0.3 |
|  | Median (IQR) dose (mg) | 250 (275) | 300 (300) | 0.5 |
| Antipsychotic (Haloperidol equivalent) treatment | No. (%) | 10 (27.8%) | 21 (18.8%) | 0.2 |
|  | Median (IQR) dose (mg) | 9.5 (14.2) | 5 (7.8) | 0.6 |
| Anticonvulsant treatment ^a, b^ | No. (%) | 3 (8.3%) | 10 (8.0%) | 0.99 |
| Antibiotic treatment ^a, c^ | No. (%) | 1 (2.8%) | 12 (10.7%) | 0.2 |
| Dexmedetomidine treatment ^a^ | No. (%) | 4 (11.1%) | 7 (6.3%) | 0.5 |
|  | Median (IQR) dose (mcg/kg/hr) | 3.4 (15.9) | 3.2 (21.1) | 0.6 |
| AWS complications | Delirium Tremens | 0 (0%) | 3 (2.7%) | 0.99 |
|  | Alcohol withdrawal seizures | 0 (0%) | 5 (4.5%) | 0.3 |
|  | Elevated liver enzymes ^d^ | 1 (2.8%) | 15 (13.4%) | 0.1 |
| Post-hospitalization mortality | No. (%) | 1 (2.9%) | 3 (3.1%) | 0.99 |
|  | Mean (±SD) duration (years) between 1^st^ hospitalization and death | 2.2 | 1.1 (1) | 0.3 |
| Laboratory results | Alkaline phosphatase (n=89, Pre-pandemic/During-pandemic= 26/63), [Median (IQR)] | 80 (45.5) | 79 (38) | 0.3 |
|  | ALT (n=94, Pre-pandemic/During-pandemic =26/68), [Median (IQR)] | 17 (20) | 19 (14) | 0.5 |
|  | AST (n=94, Pre-pandemic/During-pandemic =26/68), [Median (IQR)] | 26 (10.5) | 27 (18.8) | 0.6 |
|  | Platelet count (n=118, Pre-pandemic/During-pandemic =29/89), [Mean (SD)] | 277 (74) | 269 (70) | 0.5 |

AWS= Alcohol withdrawal syndrome

ALT= Alanine transaminase

AST= Aspartate transaminase

ICU=Intensive care unit

LOS=Length of stay

^a^ Over the whole hospital LOS

^b^ Anticonvulsants include Lamotrigine, Levetiracetam, Phenobarbital, Zonisamide

^c^ Antibiotics include Ceftriaxone, Levofloxacin, Piperacillin-Tazobactam, Vancomycin

^d^ ALT> 55 U/L and/or AST> 48 U/L (the percentage was calculated in a total of 94 patients who had laboratory values of ALT and AST).
